# Perforation of the esophagus due to thermal injury after laparoscopic radiofrequency ablation for hepatocellular carcinoma: a case for caution

**DOI:** 10.1186/s40792-018-0534-0

**Published:** 2018-10-10

**Authors:** Taishi Yamane, Katsunori Imai, Naoki Umezaki, Takanobu Yamao, Takayoshi Kaida, Shigeki Nakagawa, Yo-ichi Yamashita, Akira Chikamoto, Takatoshi Ishiko, Hideo Baba

**Affiliations:** Department of Gastroenterological Surgery, Graduate School of Life Sciences, Kumamoto University, 1-1-1 Honjo, Kumamoto, 860-8556 Japan

**Keywords:** Esophageal perforation, Radiofrequency ablation, Hepatocellular carcinoma

## Abstract

**Background:**

Several reported complications associated with radiofrequency ablation for liver tumors are due to thermal damage of neighboring organs. We herein report a first case of esophageal perforation due to thermal injury of laparoscopic radiofrequency ablation (RFA).

**Case presentation:**

A 75-year-old woman was treated repeatedly with RFA (percutaneous and laparoscopic) and transcatheter arterial chemoembolization for hepatocellular carcinoma. One week after laparoscopic RFA for recurrent HCC located in segment 2 of the liver, dysphagia and thoracic pain occurred. Upper gastrointestinal endoscopy revealed a perforated esophageal ulcer at the esophago-gastric junction. Inflammation was localized because of severe intra-abdominal adhesion due to repeat surgery, so we decided to treat the patient conservatively. The perforation of the esophagus gradually scarred, and exacerbation did not occur after restarting oral intake.

**Conclusions:**

When patients with a history of abdominal surgery or intra-abdominal inflammation undergo thermal ablation therapy, caution is required, as there is a possibility of thermal injury of unexpected organs.

## Background

The incidence of hepatocellular carcinoma (HCC) is increasing worldwide, making HCC one of the most common malignant tumors [1, 2]. Radiofrequency ablation (RFA) is now widely used to treat HCC, as it is minimally invasive, effective, easily repeated, and relatively safe. Several clinical studies have shown that RFA can achieve an overall survival rate similar to that of surgical resection in patients with small HCC [3]. However, some serious RFA complications, such as a liver abscess, intraperitoneal hemorrhaging, biloma, ground pad burn, diaphragmatic injury, pneumothorax, pleural effusion, bowel perforation, hepatic infarction, renal infarction, and tumor seeding, have been reported [4]. We herein report the first case of esophageal injury due to laparoscopic RFA.

## Case presentation

A 75-year-old woman was admitted to our hospital for the treatment of recurrent HCC located in segment 2 of the liver (S2). The patient had been diagnosed with hepatitis B virus-related liver cirrhosis 17 years earlier and with HCC (S2 and S7) 8 years earlier. Since then, RFA (percutaneous [four times] and laparoscopic [two times]) and transcatheter arterial chemoembolization (TACE; seven times) had been performed repeatedly for HCC. During laparoscopic RFA for recurrent S2 HCC, the left lateral lobe of the liver was mobilized. For recurrent HCC located in S2 of the liver, TACE was repeatedly performed; however, the therapeutic response was insufficient, and the patient was referred for further treatment.

**Fig. 1:**
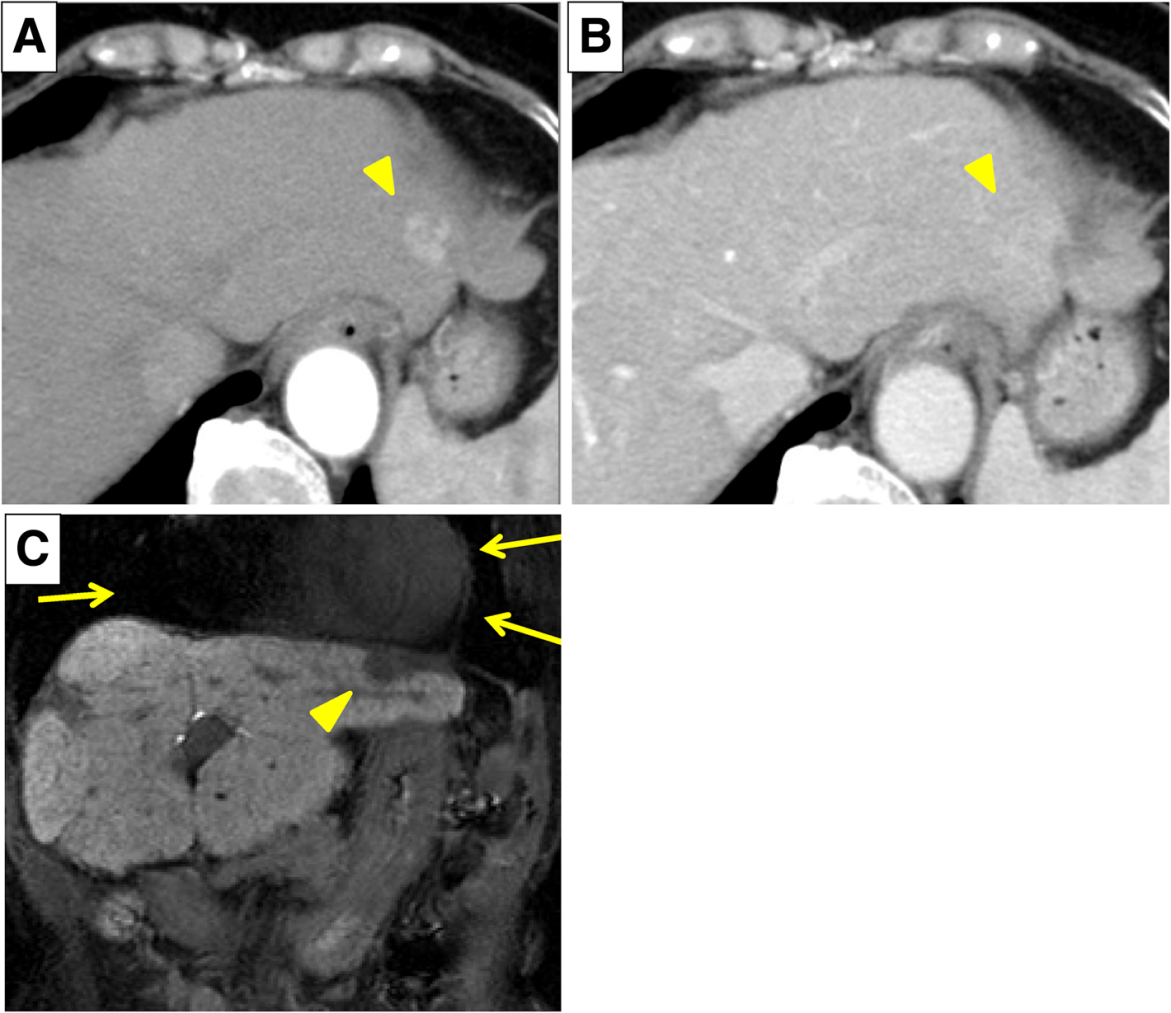
Abdominal enhanced computed tomography prior to laparoscopic RFA. There was a 1.5-cm mass in the left lateral lobe of the liver (arrow head). **a** arterial phase, **b** portal phase, and **c** ethoxybenzyl diethylenetriamine-enhanced magnetic resonance imaging prior to laparoscopic RFA. The tumor (arrow head) was located just below the heart (arrows)

Abdominal enhanced computed tomography (CT) showed a 1.5-cm mass in the left lateral lobe of the liver (S2) with arterial phase enhancement followed by washout in the portal phase (Fig. 1a, b). A laboratory analysis provided the following results: serum levels of α-fetoprotein (AFP), 124.9 ng/mL; des-γ-carboxy prothrombin (DCP), 29 mAU/mL; platelet count, 106 × 10^3^/μL; serum aspartate aminotransferase, 39 U/L; alanine aminotransferase, 15 U/L; total bilirubin, 1.2 mg/dL; albumin, 3.6 g/dL; and prothrombin time, 13.6 s. The indocyanine green retention rate at 15 min (ICG-R15) was 30.7%, and the ratio of the ^99m^Tc-galactosyl human serum albumin (GSA) scintigraphy taken up by the liver to that taken up by the liver plus heart at 15 min (LHL 15) was 0.86. The Child-Pugh score was Grade A, and liver damage, as defined by the Liver Cancer Study Group of Japan [5], was Grade B.

**Fig. 2:**
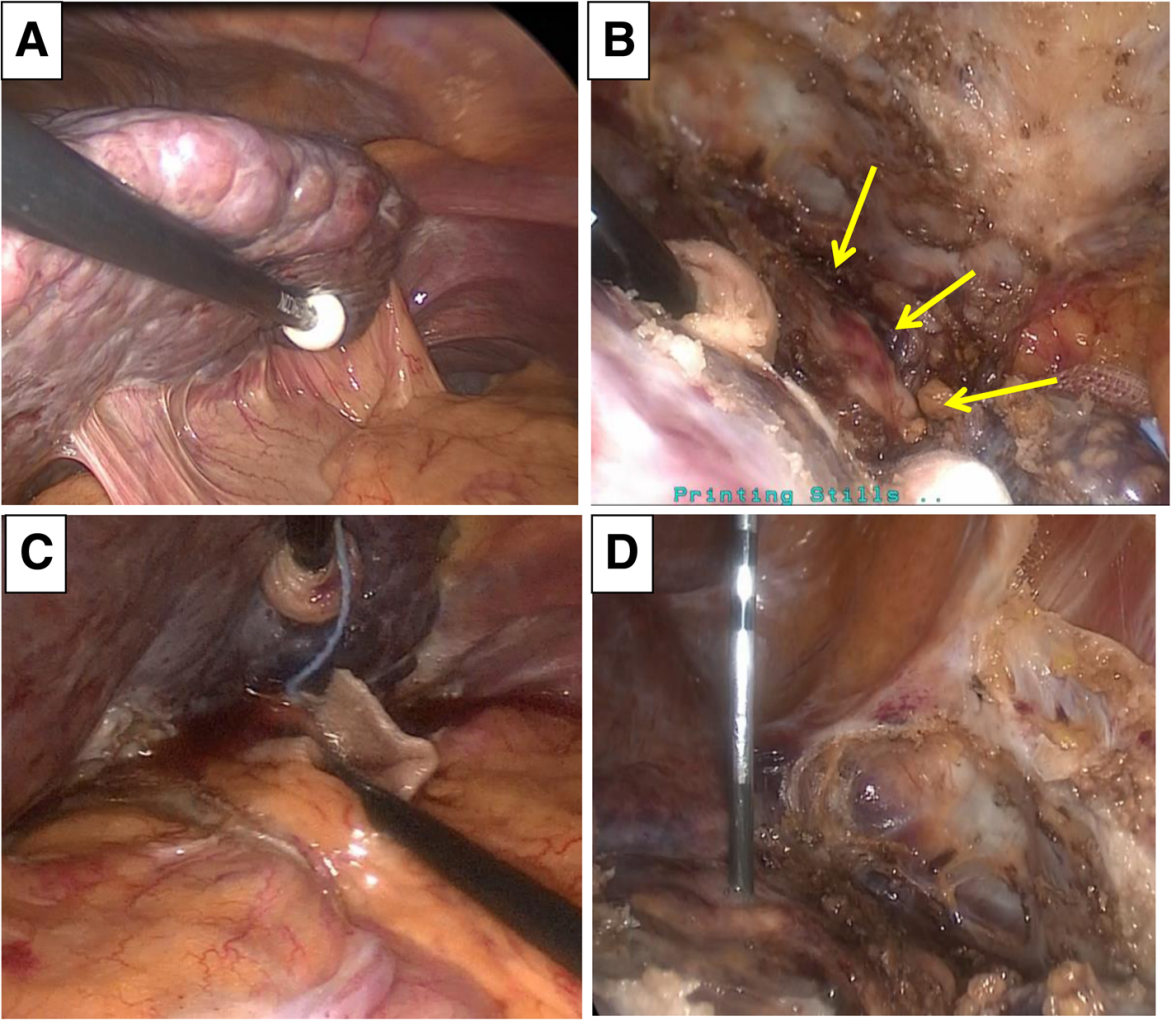
RFA laparoscopic procedure. **a** The left lateral lobe of the liver adhered severely to the diaphragm, stomach, and lesser omentum. **b** The HCC tumor was located just below the heart (arrows). **c** Gauze and some water were placed between the stomach and left lateral lobe of the liver to avoid thermal injury of the stomach. **d** A cool-tip needle was inserted vertically into the liver to avoid injuring the heart

Given that the liver function was severely impaired and the patient had already undergone RFA and TACE several times, we decided to perform RFA, not liver resection, for the treatment of this TACE-refractory small HCC. Because the tumor was adjacent to the heart (Fig. 1c), percutaneous RFA was considered to carry a high risk, laparoscopic RFA was selected. The left lateral lobe of the liver had adhered severely to the diaphragm, stomach, and lesser omentum due to mobilization of this portion during a previous session of laparoscopic RFA (Fig. 2a). We peeled away those adhesions and observed the S2 HCC tumor just below the heart (Fig. 2b). To avoid thermal injury of the stomach and heart, we first mobilized the left lateral lobe and made space between the tumor and the heart. We then placed gauze and some water between the stomach and left lateral lobe of the liver to prevent thermal injury of the neighboring organs. After observation of the tumor and liver parenchyma using a laparoscopic ultrasonography (US), we inserted a 2-cm cooled-tip needle (Radionics, Burlington, MA, USA) vertically into the liver to prevent heart injury under visual guidance, as the tumor was detected on the surface of the liver (Fig. 2c, d). After the insertion of the electrode into the lesion, we confirmed the position of the needle by US and started ablation, increasing the power to 60 W and then 100 W. The duration of the ablation (at 100 W) was 8–10 min, and the temperature was closely monitored. During the procedure, there were no major complications.

**Fig. 3:**
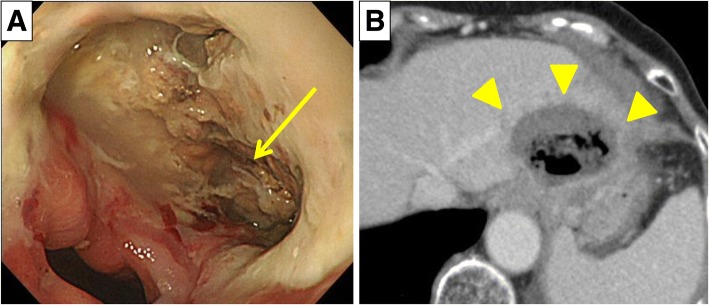
**a** Upper gastrointestinal endoscopy revealed perforation of the esophagus, and an ablated area of the liver was observed via the hole (arrow). **b** Contrast-enhanced computed tomography revealed that the cavity was localized due to severe adhesion (arrow heads)

**Fig. 4:**
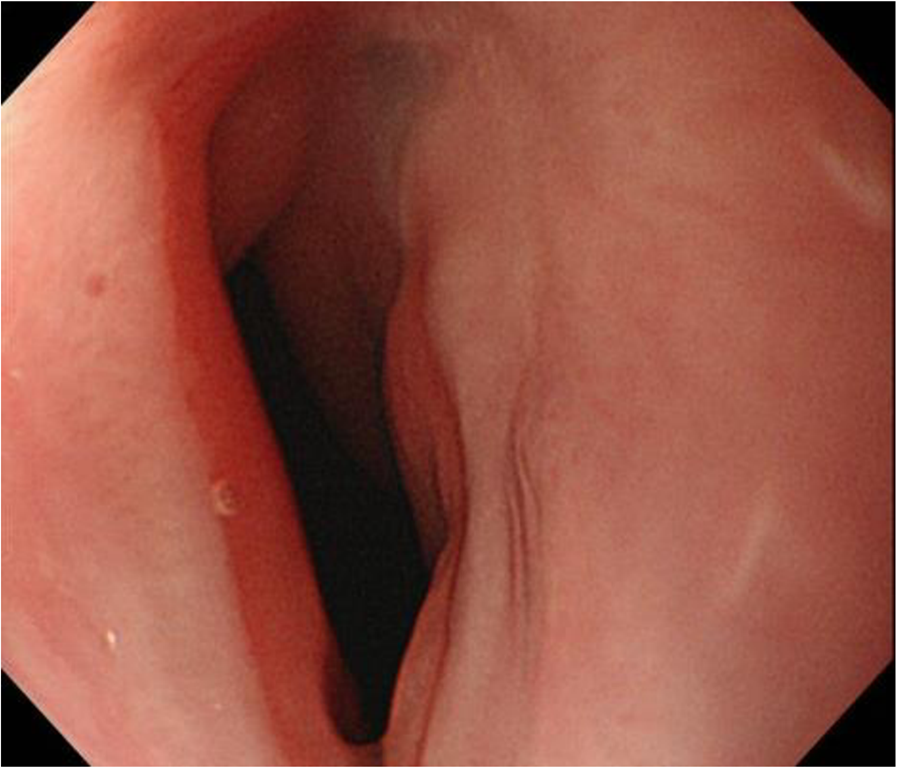
Although the patient required balloon dilatation for esophageal stenosis that developed during the healing process, the perforation was cured conservatively

At 1 week after the treatment, the patient complained of dysphagia and thoracic pain. Upper gastrointestinal endoscopy revealed a perforated esophageal ulcer at the esophago-gastric junction, and the liver could be directly seen through the perforated ulcer (Fig. 3a). Contrast-enhanced CT revealed localized free air between the left lateral lobe of the liver and the esophagus (Fig. 3b). Laboratory data revealed no exacerbation of the inflammatory response, such as via an elevated white blood cell or neutrophilic leukocyte count or C-reactive protein levels. Because inflammation was localized due to the severe intra-abdominal adhesion, we decided to treat the patient conservatively with fasting, administration of proton pump inhibitors and antibiotics, and enteral nutrition via a nasogastric tube. The perforated region of the esophagus gradually scarred over, and exacerbation was not observed after the initiation of oral intake. After discharge from our hospital, the patient complained of difficulty swallowing, and upper gastrointestinal endoscopy revealed esophageal stenosis. Although the patient later required balloon dilatation to treat the esophageal stenosis, the perforation was cured conservatively (Fig. 4), and there has been no evidence of HCC recurrence.

## Discussion

Current treatments for HCC include hepatic resection, liver transplantation, and thermal ablation such as RFA, microwave coagulation, cryotherapy, TACE, and sorafenib [6]. Among these approaches, RFA is considered to be one of the most efficient treatments for small HCC tumors. However, some serious complications associated with RFA have been reported [4]. A large cohort study including 20 institutions from Japan reported the surgical outcomes in 13,283 patients with 16,346 lesions from 1999 to 2010 [7]. Five patients (0.038%) died, including two from intraperitoneal hemorrhaging, and one each from hemothorax, severe acute pancreatitis, and perforation of the colon. In 16,346 treated nodules, 579 complications (3.54%) were observed, including 78 cases of hemorrhaging (0.477%), 276 hepatic injuries (1.69%), 113 extrahepatic organ injuries (0.961%), and 27 cases of tumor progression (0.17%). Extrahepatic organ injuries included those of the heart, lung, gastrointestinal tract, gallbladder, diaphragm, and skin. A total of nine gastrointestinal tract injuries occurred (0.055%), including three at the stomach, two at the duodenum, one at the colon, and three other gastrointestinal injuries. To our knowledge, there have been no reports of esophageal injury after RFA.

The management of recurrent HCC requires a multidisciplinary approach. Although repeated hepatectomy is the mainstay treatment for recurrent HCC, only a minority (30–35%) of patients have resectable disease because of a limited liver function reserve due to previous surgery, underlying chronic liver disease and multifocality of tumor recurrence [8, 9]. The treatment of unresectable recurrent HCC relies on loco-regional therapies for local tumor control, such as TACE and RFA. In the present case, hepatectomy was not possible because of the patient’s impaired liver function due to liver cirrhosis; the ICG-R15 was 30.7%. The S2 HCC lesion was unable to be controlled by two rounds of TACE treatment, so we selected RFA for local tumor control. Isolating the lesion from adjacent critical structures may be easier with a laparoscopic approach, potentially resulting in better control of bleeding [10]. In the present case, the tumor was located on the surface of the liver and adjacent to the heart, so we performed laparoscopic RFA to avoid heart injury. Several cases of hemorrhagic cardiac tamponade secondary to RFA have been reported [11].

A laparoscopic approach to thermal ablation has been reported to be safe and effective for small HCC [12, 13]. This approach can provide access to tumors with a “difficult” location, such as those located in the hepatic dome or adjacent to other organs, including the gallbladder, diaphragm, stomach, and colon. In our institution, percutaneous RFA was considered to be contraindicated for tumors located on the surface of the liver from the viewpoint of avoiding tumor cell dissemination. When performing laparoscopic RFA, we insert the RFA needle into the lesion under either US or direct visual guidance. During ablation, we always observe the location of the needle by laparoscopic US. Although intraoperative US is crucial for laparoscopic RFA, needle insertion under US guidance calls for skill and experience, because adjustment of the directions of the needle and US image are sometimes difficult.

In the present case, the left lateral lobe of the liver adhered severely to the diaphragm, stomach, and lesser omentum due to the previous treatment. We peeled away the adhesion, but sufficient mobilization was not obtained because of the severe adhesion and the existence of the left inferior phrenic vein in the adhered diaphragm. Because we were focused on avoiding burn injury to the heart and stomach, we failed to pay appropriate attention to the esophagus. During the procedure, although we always paid attention to the position of the needle and other organs, including the diaphragm, heart, and stomach by ultrasonography, we did not recognize that the esophagus was located close to the ablated area. We should have paid closer attention to neighboring organs, especially those that are not usually considered in such cases, such as the esophagus. Patients with a history of abdominal surgery or intra-abdominal inflammation should be regarded as being at high risk of thermal injury of unexpected organs.

Perforation of the digestive tract does not always require surgical intervention. In the present case, CT findings and laboratory data suggested that inflammation was localized due to severe intra-abdominal adhesion. Therefore, we decided to treat the patient conservatively. However, of note, if conservative management is selected, careful observation is required, and surgical intervention should be performed if signs of spreading of inflammation become apparent.

## Conclusions

We describe a case of esophageal perforation due to thermal injury of laparoscopic RFA. When patients with a history of abdominal surgery or intra-abdominal inflammation undergo thermal ablation therapy, caution is required, as there is a possibility of thermal injury of unexpected organs.

## Abbreviations

AFP: α-Fetoprotein
CT: Computed tomography
DCP: Des-γ-carboxy prothrombin
HCC: Hepatocellular carcinoma
RFA: Radiofrequency ablation
TACE: Transcatheter arterial chemoembolization
US: Ultrasonography
